# Patient Preferences and Willingness-To-Pay for a Home or Clinic Based Program of Chronic Heart Failure Management: Findings from the Which? Trial

**DOI:** 10.1371/journal.pone.0058347

**Published:** 2013-03-07

**Authors:** Jennifer A. Whitty, Simon Stewart, Melinda J. Carrington, Alicia Calderone, Thomas Marwick, John D. Horowitz, Henry Krum, Patricia M. Davidson, Peter S. Macdonald, Christopher Reid, Paul A. Scuffham

**Affiliations:** 1 Centre for Applied Health Economics, School of Medicine, Griffith Health Institute, Griffith University, Meadowbrook, Queensland, Australia; 2 NHMRC Centre of Research Excellence to Reduce Inequality in Heart Disease Preventative Health, Baker IDI Heart and Diabetes Institute, Melbourne, Victoria, Australia; 3 Cleveland Clinic, Cleveland, Ohio, United States of America; 4 The Queen Elizabeth Hospital, Adelaide, South Australia, Australia; 5 University of Adelaide, Adelaide, South Australia, Australia; 6 Centre of Cardiovascular Research & Education in Therapeutics, School of Public Health and Preventative Medicine, Monash University, Melbourne, Victoria, Australia; 7 University of Technology, Sydney, New South Wales, Australia; 8 St Vincent’s Hospital, Sydney, New South Wales, Australia; 9 Victor Chang Cardiac Research Institute, Sydney, New South Wales, Australia; 10 University of New South Wales, Sydney, New South Wales, Australia; The University of Hong Kong, Hong Kong

## Abstract

**Background:**

Beyond examining their overall cost-effectiveness and mechanisms of effect, it is important to understand patient preferences for the delivery of different modes of chronic heart failure management programs (CHF-MPs). We elicited patient preferences around the characteristics and willingness-to-pay (WTP) for a clinic or home-based CHF-MP.

**Methodology/Principal Findings:**

A Discrete Choice Experiment was completed by a sub-set of patients (n = 91) enrolled in the WHICH? trial comparing home versus clinic-based CHF-MP. Participants provided 5 choices between hypothetical clinic and home-based programs varying by frequency of nurse consultations, nurse continuity, patient costs, and availability of telephone or education support. Participants (aged 71±13 yrs, 72.5% male, 25.3% NYHA class III/IV) displayed two distinct preference classes. A latent class model of the choice data indicated 56% of participants preferred clinic delivery, access to group CHF education classes, and lower cost programs (p<0.05). The remainder preferred home-based CHF-MPs, monthly rather than weekly visits, and access to a phone advice service (p<0.05). Continuity of nurse contact was consistently important. No significant association was observed between program preference and participant allocation in the parent trial. WTP was estimated from the model and a dichotomous bidding technique. For those preferring clinic, estimated WTP was ≈AU$9-20 per visit; however for those preferring home-based programs, WTP varied widely (AU$15-105).

**Conclusions/Significance:**

Patient preferences for CHF-MPs were dichotomised between a home-based model which is more likely to suit older patients, those who live alone, and those with a lower household income; and a clinic-based model which is more likely to suit those who are more socially active and wealthier. To optimise the delivery of CHF-MPs, health care services should consider their patients’ preferences when designing CHF-MPs.

## Introduction

Chronic heart failure (CHF) is a burdensome condition associated with a high mortality rate and substantial health care costs [1]–[3]. Multidisciplinary programs assisting patients and their families to manage their CHF have been shown to improve quality of life and survival, as well as to reduce hospital readmission rates and the costs associated with the management of the condition [4]. Consequently, CHF management programs (CHF-MPs) are now part of the gold-standard management of CHF following an acute hospital admission [5].

Providing care that is consistent with patient values is a key consideration in building a partnership between patients and health care providers, and is an integral component of patient-centred health care [6]. There is a substantial treatment burden borne by CHF patients, and this is contributed to by factors related to the provision of services to assist with the management of CHF [7]. Further, there is large variation in the design of CHF-MPs. One design consideration is the mode of delivery, with models of care including face-to-face programs delivered either at a hospital clinic or in the patient’s own home [8]. This was the focus of the WHICH? Trial (Which Heart failure Intervention is most Cost-effective & consumer friendly in reducing Hospital care; Australian New Zealand Clinical Trials Registry number 12607000069459 (http://www.anzctr.org.au) [9]. Although there was no difference in the primary endpoint (risk of death or unplanned hospitalisation during 18 month follow-up), the home-based intervention arm was associated with around one third less recurrent hospital days stay and significantly reduced total health care expenditure [10]. However, beyond consideration of pure cost-benefits, understanding patient perspectives, preferences and choices for the delivery of programs for the management of CHF is a crucial contextual factor to be considered alongside evidence showing the clinical and cost-effectiveness of these programs; particularly as patient preferences are likely to be a key component in optimising program uptake [6].

Program participation, persistence and adherence to recommendations are likely to be substantially greater when patients are provided with their preferred choice for delivery of the program. What is important in understanding preferences around disease MPs and other healthcare interventions is the trade-offs patients are prepared to make in order to have their preferred option. Understanding these trade-offs provides insight in to the strength of the preference and enables design of interventions that will optimise the allocation and use of scarce healthcare resources. Further, understanding how much more a patient is prepared to pay to have their preferred choice allows us to quantify the strength of their preference; this can be used alongside other attributes of the program to identify the key factors of relevant importance in any CHF-MP or other healthcare intervention.

Within the WHICH? Trial comparing a clinic versus home-based CHF-MP [9], therefore, we prospectively aimed to elicit patient preferences for the delivery of the two forms of face-to-face CHF-MP, in order to identify:

1. Preferences for program characteristics including associations with patient characteristics; and,
2. The value of home and clinic-based program delivery by estimating patients’ willingness to pay (WTP) for their preferred delivery mode (home or clinic).

## Methods

The investigation conformed to the principles outlined in the declaration of Helsinki. The preference study was approved as a sub study of the parent WHICH? trial by the Human Research Ethics Committees of the Princess Alexandra Hospital, Brisbane; St Vincent’s Hospital, Sydney; the Queen Elizabeth Hospital, Adelaide; and Griffith University, Queensland, Australia. All participants were provided with an information sheet, had any questions answered by the research nurse, and provided written informed consent. Participation was completely voluntary, and potential participants who declined to participate or otherwise were eligible for all routine health care treatment and services and were not disadvantaged in any other way by not participating in the study.

A discrete choice experiment (DCE) was employed to assess patient preferences for program delivery. The DCE is a choice-based stated preference method for quantifying preferences and has the potential to estimate the uptake of a program in a population as well as to place an economic value on a service, by estimating participants’ willingness to pay. The DCE has a firm theoretical basis in random utility theory and Lancaster’s theory of value [11]–[13]. Advantages of the DCE as a choice based method that requires participants to explicitly make trade-offs between the characteristics of a program in decision-making have led to it being a popular method for eliciting preferences for healthcare [14], with research demonstrating it’s potential in assessing a patient-centred approach to service delivery [15].

### Development of the DCE Instrument

A DCE instrument was designed to assess patient preferences around the delivery of a CHF-MP. Participants were asked to make repeated hypothetical choices between a clinic-based and home-based program. Each program was described according to five attributes (Table 1) which were predominantly developed based on a qualitative study comprising semi-structured interviews with twelve CHF patients [16]. This allows an assessment of the relative impact of each of these attributes on the overall program preference. Each attribute had two levels; for four attributes the levels were generic across programs, whilst for the fifth attribute (cost) the levels differed between the clinic and home alternatives.

**Table 1:** Attributes and levels.

Attribute	Description and levels
**How often you see** **the nurse**	• **Once every week:** You see the nurse once each week
	• **Once every month:** You see the nurse once each month
**Continuity of contact**	• **Same nurse:** You usually see the same nurse each time
	• **Different nurse:** You often see a different nurse each time
**Cost to you each time** **you see the nurse**	**[For home program]:** This describes the cost to you personally of seeing the nurse, which you would be asked to pay from your own pocket.
	• **$0:** There is no charge each time you see the nurse.
	• **$15:** We would like you to imagine you are asked to pay $15 from your own pocket each time you see the nurse. You will not have to pay this amount. This is just a way of finding out how strongly you feel about heart failure management programs.
	**[For clinic program]:** If you see a nurse at a clinic, you might have to pay travel costs to get to the clinic (for example, you might have to pay for a bus, taxi, or car-parking).
	• **You pay:** You pay your own costs to travel to the clinic each time you see the nurse.
	• **$15 voucher:** We would like you to imagine that you are given a $15 voucher to pay for your travel costs each time you see the nurse.
	If you see a nurse at home, you would not need to pay travel costs (**$0**), as you would not need to travel to see the nurse.
**Telephone advice** **service**	• **Yes:** You have access to a telephone number which you can call to speak to a nurse if you need advice about your heart failure. There is no additional charge for this service.
	• **No:** You do not have access to this telephone service.
**Group education class**	• **Yes:** You are offered group education classes where you can learn more about your heart failure. These are run at a local clinic and usually involve some information sessions provided by nurses, doctors, a pharmacist, a dietician, and a physiotherapist. Often, these programs provide an opportunity to meet other people with heart failure. There is no additional charge for this service.
	• **No:** You do not have the opportunity to attend group education classes.

**Figure 1:**
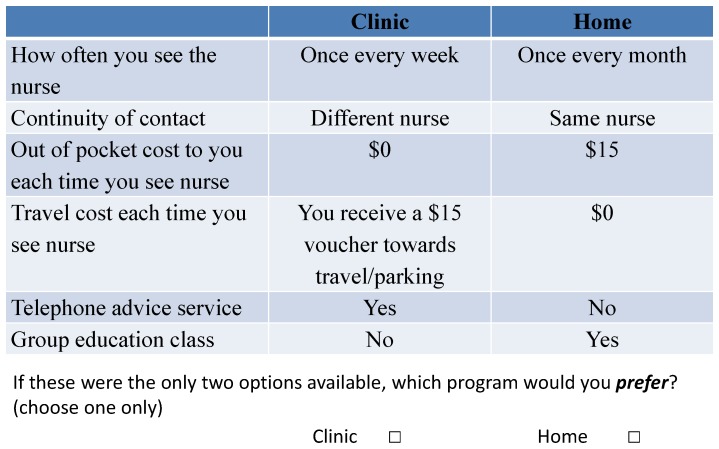
Sample choice set.

The level for each attribute varied across alternative programs according to a D_z_-efficient fractional factorial experimental design, estimated using NGENE software (version 1.0.2, 2010). This ensured optimal statistical power for the design. The design identified 20 choice sets. To ensure the number of choices faced by this relatively elderly and frail population was manageable, these were divided into 4 blocks of 5 choice sets, with participants randomised to one of the four blocks. A sample choice set is shown in Figure 1. In addition to the choice sets, participants were asked to indicate their direct preference for clinic or home independent of the program characteristics (“If you could only choose clinic or your home, which would you choose?”), their WTP for their preferred program (clinic or home), socio-demographic characteristics, and their self-reported travel costs to attend a clinic at their local hospital (even if they have never seen a nurse at the clinic).

### Study Participants and Data Collection

Approximately 70% of health expenditure in Australia is funded by the government, with the Australian Government responsible for most community based services and State Governments responsible for hospital and outpatient services. Whilst State hospital services are generally provided at no cost to the patient, approximately half of the population have private health insurance [17] and there can be substantial out-of-pocket costs for many community services regardless of insurance status.

Following a hospitalisation for CHF, the WHICH? trial randomised participants to receive a CHF-MP delivered either in their own home via an outreach program or via a specialist CHF outpatient clinic [9]. Patients were recruited from three major tertiary hospitals funded by State Government health services in Australia and, as described in more detail previously [9], care was standardised based on evidence-based elements of multidisciplinary care [18].

The DCE instrument was administered face-to-face by research nurses at the final WHICH? Trial follow-up appointment (12 to 18 months post enrolment) between July 2010 and March 2011. A total of 126 consecutive trial participants due to attend their final follow-up appointment were invited to participate. Visual aids and cards containing the choice sets were available to support survey administration. The survey instrument was pilot tested in the first 12 participants. Since no amendments were required to the instrument, the data for these 12 participants was included in this final analysis.

### Data Analysis

Participant characteristics were compared using the Chi^2^ test for categorical and independent t-test for continuous variables. The choice data were analysed using a latent class (LC) model estimated using the statistical software package NLOGIT (version 4.0.1, 2007). The LC model allows preferences for program delivery to vary between participants and an assessment of associations between participant characteristics and preference strength [19], [20]. The LC model was specified with two classes. To explore associations with class membership, respondent characteristics were entered into the model one at a time; any characteristic that (i) was significant in explaining class membership at the 10% level and (ii) resulted in an equal or improved fit as measured by the Akaike Information Criterion (AIC), was retained for the final model specification.

For ease of interpretation, the cost attribute was coded continuously. For the home alternative the out-of-pocket cost was coded for this variable as $0 or $15 (AU$1 ≈ US$1≈ Euro 0.8 at 1^st^ July 2012). Due to the potential for out of pocket costs in the Australian health system, the inclusion of a possible out of pocket cost in the design was considered to be realistic for the home-based CHF-MP. For the travel alternative, travel compensation ($0 or $15) was converted to a travel cost adjusted for the sample’s mean self-reported travel cost ($10.82) and coded for this variable as $-4.18 (where a $15 voucher was provided) or $10.82 (where the participant was told they had to pay their own travel costs). All other attributes were effects coded in the model [21].

To address the second objective, WTP for program delivery was estimated using two different methods: indirectly from the LC model of choice responses using the compensating variation method [22], and using direct responses to a dichotomous WTP bidding question in the survey. In the bidding approach, participants were asked to indicate their WTP using a dichotomous response (yes/no) to a bid, with the bid varied in a ping pong fashion according to a bidding algorithm until the respondent stated they would not pay the offered amount. At this point the last value the respondent agreed they would pay was used to estimate the mean and median marginal WTP for the preferred delivery mode. To minimise any starting point bias [23], participants were randomised to a starting bid of either AU$10 or AU$20. The use of two methods to estimate WTP allows data triangulation and potentially an increased level of confidence around the findings.

## Results

Of the 126 trial participants invited to participate in the DCE, 97 were judged by the research nurse to be cognitively able and agreed, representing a 77% response rate. Two participants who were not randomised to a survey version in error and four participants with missing choice data were excluded; thus, the choice model was based on 455 choice observations (5 choice sets from each of the 91 respondents).

Table 2 summarises the respondent characteristics. With the exception of more male individuals, participants in the preference survey were typical of CHF patients. Their socio-demographic and clinical profile did not significantly differ between trial groups or from the remainder of surviving patients in the study (p>0.05; comparative data not available for income, employment or health insurance status). Respondents were more cognitively intact than non-participants (p<0.05) likely reflecting the inclusion requirements for the preference survey. Participants were approximately evenly distributed between each trial arm (51.6% clinic, 48.4% home), DCE block, and WTP starting bid. The mean estimated travel cost from home to clinic reported by all participants (regardless of whether they were receiving a clinic or home based service) was $10.82 (SD 11.68).

**Table 2:** Respondent characteristics by trial randomisation group.

	Respondentcharacteristics		Clinic-basedCHF-MP(N = 47)	Home-basedCHF-MP(N = 44)	Allrespondents(N = 91*)
**At entry to WHICH? trial:**	Site (for WHICH?)†	New South Wales	23 (48.9%)	18 (40.9%)	41 (45.1%)
		Queensland	10 (21.3%)	8 (18.2%)	18 (19.8%)
		South Australia	14 (29.8%)	18 (38.3%)	32 (35.2%)
	Age	Mean (SD) Yrs	71.00 (14.13)	70.11 (11.18)	70.57 (12.72)
	Gender	Male	34 (72.3%)	32 (72.7%)	66 (72.5%)
	Ethnicity	European/Caucasian	43 (91.5%)	42 (95.5%)	85 (93.4%)
		Aboriginal/Torres Strait Islander	2 (4.3%)	1 (2.3%)	3 (3.3%)
		Asian	2 (4.3%)	1 (2.3%)	3 (3.3%)
	Marital status	Married/with partner	20 (42.6%)	17 (38.6%)	37 (40.7%)
		Widowed	11 (23.4%)	6 (13.6%)	17 (18.7%)
		Separated/Divorced	10 (21.3%)	11 (25%)	21 (23.1%)
		Never married	6 (12.8%)	10 (22.7%)	16 (17.6%)
	Highest education	Primary school	6 (13.0%)	9 (21.4%)	15 (17.0%)
		Secondary school	18 (39.1%)	19 (45.2%)	37 (42.0%)
		TAFE/Trade school	16 (34.8%)	7 (16.7%)	23 (26.1%)
		Degrees, Diploma or Graduate Certificate	6 (13.0%)	7 (16.7%)	13 (14.8%)
	Distance from home to clinic	Mean (SD) kms	9.23 (10.32)	10.07 (15.12)	9.64 (12.80)
	HF duration prior to WHICH?trial enrolment	Mean (SD) mths	34.47 (55.03)	36.27 (70.99)	35.34 (62.90)
**At completion of DCE** **survey (final follow-up):**	Main source of income	(self-) Employed	4 (8.9%)	6 (13.6%)	10 (11.2%)
		Supported by family member	2 (4.4%)	0 (0%)	2 (2.2%)
		Pension from Government	33 (73.3%)	32 (72.7%)	65 (73.0%)
		Self-funded retiree	4 (8.9%)	3 (6.8%)	7 (7.9%)
		Other	2 (4.4%)	3 (6.8%)	5 (5.6%)
	Income (annual household, AU$)	Up to $25,000	28 (68.3%)	31 (79.5%)	59 (73.8%)
		$25,0001–$50,000	6 (14.6%)	5 (12.8%)	11 (13.8%)
		$50,0001–$75,000	2 (4.9%)	2 (5.1%)	4 (5.0%)
		$75,0001–$100,000	3 (7.3%)	1 (2.6%)	4 (5.0%)
		$100,0001–$125,000	1 (2.4%)	0 (0%)	1 (1.3%)
		>$125,000	1 (2.4%)	0 (0%)	1 (1.3%)
	Health Insurance	Hospital +/− Extras	15 (31.9%)	13 (29.5%)	28 (31.5%)
	MOCA	Mean (SD)	25.14 (3.61)	25.26 (3.38)	25.20 (3.48)
	NYHA	II	33 (70.2%)	35 (79.5%)	68 (74.7%)
		III	14 (29.8%)	9 (20.5%)	23 (25.3%)
	EQ5D	Mean (SD)	0.76 (0.18)	0.75 (0.22)	0.75 (0.20)
	Estimated travel cost to clinic	Mean (SD) $	12.88 (13.66)	8.63 (8.63)	10.82 (11.68)
**Survey version** **completion:**	DCE block	1	9 (19.1%)	11 (25.0%)	20 (22.0%)
		2	11 (23.4%)	14 (31.8%)	25 (27.5%)
		3	14 (29.8%)	12 (27.3%)	26 (28.6%)
		4	13 (27.7%)	7 (15.9%)	20 (22.0%)
	WTP starting bid	$10	21 (44.7%)	25 (56.8%)	46 (50.6%)
		$20	26 (55.4%)	19 (43.2%)	45 (49.4%)

DCE discrete choice experiment; WTP willingness to pay; MOCA Montreal Cognitive Assessment Tool; NYHA New York Heart Association; TAFE Tertiary and Further Education Institution.

* Proportion (%) refers to valid cases. Missing responses excluded from denominator: 5×EQ5D, 2×Employment, 11×Income, 2×Insurance, 5×MOCA, 3×Education status.

† A greater number of study participants had completed follow-up for the Queensland site prior to ethical approval being granted for the preference study and were therefore not invited to participate in the preference study; this explains the higher number completing from the other two sites.

When asked to indicate a direct preference between a clinic or home-based service, 48 (52.7%) participants stated they would prefer clinic and 43 (47.3%) would prefer home. However, there was no observed association between study arm and preference (53% of those in the clinic arm preferred home and 59% of those in the home arm preferred clinic (p = 0.24). The slightly greater preference overall for clinic-based services was reflected in the raw data observation of a higher proportion of respondents always choosing the clinic alternative across the five choice sets regardless of the level of the other DCE attributes (24, 26.4%), compared to respondents that always chose the home alternative (20, 22.0%). An exploratory analysis to identify associations between whether or not a respondent always chose the same alternative across all five choice sets and their clinical and socio-demographic characteristics (as listed in Table 2) suggested those that were married or living with a partner were less likely to have a dominant preference for home or clinic and more likely to trade between home and clinic based on the levels of the other attributes (p = 0.03). No other significant observations were observed for the raw data.

### Preferences for Program Characteristics

For the LC model, only age, being married/living with a partner and household income met the criteria for inclusion in the final LC model. None of the other tested characteristics, including study arm (clinic or home), were close to significance in predicting preference class membership (p>0.1).

The results of the LC model are summarised in Table 3. The model represents a reasonable fit for the choice data (pseudo-R^2^ of 0.296) [24]. Participants displayed two distinct classes of preference for a CHF-MP, with the program attributes having a quite different impact on preferences in each class. On average, members of class one preferred clinic over home, access to group education classes, and lower cost programs (p<0.05). However, they were indifferent to the frequency of appointments or the availability of a phone advice service. Conversely, members of class two preferred home over clinic, monthly rather than weekly visits, and access to a phone advice service (p<0.05), but were indifferent to accessing group education classes and notably were not sensitive to cost. Continuity of nurse contact was consistently important with both classes preferring to see the same nurse at each visit (p<0.05).

**Table 3:** Latent class model coefficients.

	Variable	Referent	Class 1	Class 2
**Utility parameters in** **latent class**	Constant for home	(clinic)	***−1.731	*** 2.101
	Weekly frequency	(monthly)	0.020	***−0.515
	Same nurse	(different)	** 0.232	* 0.158
	Cost		***−0.068	−0.016
	Access phone advice	(no access)	0.063	**0.196
	Access group education	(no access)	**0.334	0.160
**Class probability model**	Constant		* 2.865	0 (fixed)
	Age		*−0.036	0 (fixed)
	Married/Living with partner	(single, divorced or widowed)	0.429	0 (fixed)
	Household income (>AU$50,000)	(≤$50,000)	* 0.001	0 (fixed)
**Average class probability**			0.558	0.442
**Model statistics**	No. observations		455	
	LL		−222.105	
	AIC		1.047	
	Pseudo R^2^		0.296	

AIC Akaike Information Criterion; LL Log Likelihood.

*** p<0.01.

** p<0.05.

* p<0.1.

On average, each participant had a probability of 0.56 of belonging to class one and 0.44 of belonging to class two. Thus, the choice model predicted similar preference proportions to that stated directly by participants, supporting model validity. There was a trend for participants who were younger, married or living with a partner, or who had a higher household income, to be more likely to belong to class one (and therefore to prefer clinic) relative to class two; however, this trend failed to reach statistical significance (p = 0.09, 0.11, 0.07 respectively).

### Willingness to Pay for Preferred Program Delivery

For each latent preference class in the LC model of choice responses, the WTP was estimated for a move from a state where only the least preferred alternative was available (home for class 1 and clinic for class 2), to a state where a choice was available between clinic or home (with levels of the other attributes held constant). For each member of class 1, an average welfare gain of $9.07 was associated with the provision of a clinic program for each visit. The validity of estimating WTP for members of class 2 is compromised by the lack of significance of the cost parameter for this class in the LC model. Nevertheless, an indicative estimate from the LC model is that each member of class 2 would be willing to pay $105.40 for having a home option available.

In comparison, using the dichotomous bidding approach, those who indicated they would prefer a clinic program were willing to pay on average AU$27.28 (median $20.00, IQR $10.00–$30.00) more for a clinic visit rather than home visit. The participants who indicated they would prefer a home program were willing to pay on average AU$15.43 (median $15.00, IQR $8.75–$20.00) more for a home visit rather than clinic visit.

## Discussion

Despite the clear relevance of patient preferences to service design and delivery, few studies have explored the preferences or opinions of patients around the delivery of CHF-MPs. This study provides an example of how the inclusion of a preference study alongside a clinical trial can add an additional dimension to the evaluation of competing CHF-MPs; it shows the acceptability of different program characteristics to patients and elucidates the nuances of patient preferences for program provision. Based on a large subset of patients enrolled in the WHICH? Trial [9], [10], this study found preferences for the delivery of a program via a clinic or home based setting were largely dichotomised; approximately half of participants expressed a strong preference for either a clinic or home based program, with the other half having less strong preferences for the place of delivery. Overall, the principal finding suggests that two distinct program models would deliver the preferred services to the vast majority of patients with CHF. A home-based model with remote services (such as telephone advice) is more likely to suit older patients, those who live alone, and those with a lower household income; and a clinic-based model with on-site services such as group education classes is more likely to suit those who are more socially active and wealthier.

Regardless of the program design, the importance of continuity of staff contact was shown. Although this is aligned with previous research [7], to our knowledge this is the first time the importance of this characteristic has been objectively determined with preference weights in a DCE format. Having continuity of staff was almost as important for respondents as access to group education (class 1) or telephone advice (class 2). Those with less strong preferences were willing to trade between a home or clinic-based program dependent on the other characteristics provided by the program. If individuals who have a strong preference for a program could be identified, clinicians could focus on targeting these individuals in order to provide patients with their preferred choice of delivery. An exploratory analysis of our raw data suggests that those that are not married or living with a partner are more likely to have strong preferences for either a home or clinic-based program and could be targeted. However, the relatively small sample size may have limited our test for association and further studies exploring this association are required if a predictive model is to be developed.

A strong program preference might be expected given the nature of the trial and exposure to one or the other mode of CHF-MP. Interestingly though, we did not observe an association between preference for a clinic or home based program and participant allocation in the parent trial. The data from this preference study provide important additional information when interpreting the major findings of the WHICH? Trial – that these commonly applied forms of CHF-MP are similar in terms of event-free survival from re-hospitalisation or death (the primary endpoint), but may differ in respect to duration of recurrent hospital stay and, critically, in terms of economic considerations, with total health care expenditure in favour of the home-based intervention [10]. It is important to note that during the survey, participants were not aware of potential differences in respect to health outcomes.

This preference study can also be used to value different CHF-MPs within an economic framework. In theory, both the DCE and direct WTP methods applied in this study can provide an estimate of the economic value or welfare gain associated with program provision from a patient perspective. The directly elicited marginal WTP using a bidding algorithm provided a slightly higher estimate for a clinic visit in those who preferred clinic (median AU$20, IQR $10–$30) than for a home visit in those who preferred home (median AU$15, IQR $8.75–$20.00). Conversely, the DCE findings suggest substantially greater value for a home rather than clinic program in the cohort who responded. However, there is considerable uncertainty around the estimated WTP value for home programs for several reasons. Firstly, the cost attribute for class 2 did not reach significance, resulting in a low level of precision for the estimate. Further, the absolute size of the WTP value in this population, who were generally of low income and not employed, was surprisingly large. Even though the two WTP methods technically estimated different concepts (i.e. to estimate WTP for home, the DCE method estimates a WTP for having an option of home or clinic rather than being forced to have a clinic program, and the bidding method estimates a marginal WTP for home over clinic), a large difference in WTP between the two methods as was observed for the home-based program would seem unexpected.

Estimating WTP in the context of health care, where services are subject to public provision and patients may not ordinarily have to pay for services out of pocket, is challenging. Previous studies have reported biases with estimates for WTP directly elicited from respondents [23], and non-attendance to the cost attribute included in choice experiments [25]. It would seem possible that participants in the current study were not prepared to consider the possibility of having to pay for a home visit which would conventionally be provided at no cost in a public hospital service. It is also possible that on average membership of class 2, which was weakly associated with older age and a lower household income, may be correlated with a lower ability to understand the DCE task and the hypothetical concept of paying for a service. Nevertheless, estimating the value of the characteristics of a health service beyond those that relate directly to health outcomes is an important consideration in welfare maximisation.

Despite the uncertainty in the WTP estimates provided by this study, it is unlikely that the value derived from patients for their preferred home-based or clinic-based service would offset the substantial cost advantage to the health system in favour of home-based programs reported by the WHICH? Trial (median cost per day follow-up $52 (95%CI: $17–$140) for clinic, $34 (95%CI: $13–$81) for home, difference in medians $18 per day p = 0.034) [10]. As noted earlier, the DCE reported here did not test whether spending fewer days in hospital, as reported by the WHICH? Trial to be a beneficial outcome favouring home-based programs [10], would change the choices people made based on characteristics of survey delivery. Therefore, it is possible that some individuals preferring clinic-based delivery may change their preference if provided with this information, and WTP for a home-based program may have been underestimated. The findings of this preference sub-study and the parent trial taken together would suggest that overall, if only one service can be provided, a home-based program is likely to provide the greatest societal value.

Perceived treatment burden has been associated with poorer self-reported health outcomes and reduced treatment adherence [26], [27]. Since it is the perception of treatment burden that is important, an understanding and active consideration of patient preferences is likely to be an important intermediary in reducing treatment burden and its sequelae, particularly in the management of chronic diseases with high levels of co-morbidity such as CHF [26]. Therefore, not withstanding the apparent economic advantages associated with the home-based program, flexibility of program delivery is an important goal. Inflexible programs may lose their economic advantage and lead to suboptimal health outcomes if they do not meet patient preference and as a consequence result in high treatment burden and/or non-compliance.

The DCE in this study was undertaken at the end of a trial when all participants had experienced either a clinic or home-based program. Although we tested for an association between the trial arm and preference structure, and did not identify such an association, it is possible that the preferences of treatment naïve patients would be different. The generalisability of this preference study may potentially be limited by the sample. Whilst broadly reflecting the characteristics of surviving members of the wider trial cohort, the members of the preference sample were cognitively intact, the majority were male, and most had relatively mild symptoms of heart failure at the time they were surveyed. Further, trial participants may have been more likely than non-trial participants to accept the possibility of being allocated to either a clinic or home-based program [9]. Therefore, it is unclear how generalisable the findings of the preference survey are to the broader population of CHF patients, and further research is needed to assess the preferences of different CHF populations. Finally, a combination of the sample size and relatively high proportion of participants who were unwilling to trade between programs may limit the power of the analysis to test associations between respondent characteristics and class membership.

The strengths of this study include a patient-centred focus and the multiple methods (direct and indirect) that were employed to assess preferences and WTP. Whilst discrete choice methods have become popular to assess patient preferences for health care [14], this study represents a novel application of the DCE to assess patient preferences for a disease MP. The characteristics of delivery of a CHF-MP are important considerations for both health care providers and patients alike. This study shows that the acceptability of a CHF-MP to patients is affected by how it is delivered. To optimise the value that society receives from these programs and arguably patient adherence, it is vital to consider patient preferences for program delivery in addition to more conventional measures of clinical benefits and health system costs. Further research into patient preferences for programs to assist with the management of cardiovascular disease including heart failure and in particular hybrid models would aid our understanding of optimal service design. Incorporation of discrete choice methods to evaluate service delivery more broadly and prospectively would support a greater understanding of patient preferences in the management of CHF and other burdensome conditions.
